# A modified multiple branched graft for thoracoabdominal aortic aneurysm repair

**DOI:** 10.1186/s13019-017-0593-5

**Published:** 2017-06-05

**Authors:** Liang-Wan Chen, Xi-Jie Wu, Hua Cao, Xiao-Fu Dai

**Affiliations:** Department of Cardiac Surgery, Union Hospital, Fujian Medical University, Fuzhou, Fujian 350001 China

**Keywords:** Thoracoabdomial aortic aneurysm, Aortic surgery, A branched graft

## Abstract

**Background:**

To reduce some problems of traditional graft, we devise a modified multiple branched graft for repair of Crawford extent II and III thoracoabdominal aortic aneurysm (TAAA).

**Case presentation:**

We described a modified multiple branched graft for Crawford extent II and III thoracoabdominal aortic aneurysm (TAAA) repair in 8 patients, Which comprised a main graft and three branches, and the third branch was bifurcated into two limbs.

**Conclusions:**

Our initial experience demonstrated that this modified multiple branched graft may make the thoracoabdominal aortic aneurysm(TAAA) repair easier and safer.

## Background

In the traditional graft replacement of thoracoabdominal aortic aneurysm (TAAA), a straight graft is usually used, and several aortic patches containing the orifices of intercostal, celiac, superior mesenteric, and renal arteries are sutured to the openings in the straight graft. Recently, presewn tetrafurcate graft was used for TAAA repair, and clinical results showed this tetrafurcate graft could effectively short the ischemic time of the reimplantated visceral organs and facilate anastomotic hemostatisis [1, 2]. Therefore, this technique could be an attractive alternative to traditional straight graft for TAAA repair. Because this tetrafurcate graft was originally designed for replacement of the aortic arch, it had not any branch suitable for attachment to the vital intercostal arteries. Furthermore, the directions of the branches were not conformed to the directions of the celiac, superior mesenteric, and renal arteries, so these branches should be usually longer than we expected for keeping them from being twisted or kinking. To reduce such problems, in this report we represent a modified multiple branched graft for repair of Crawford extent II and III TAAA.

## Case presentation

**Fig. 1:**
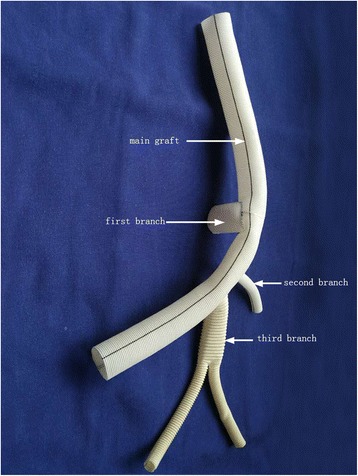
The modified multiple branched graft

The modified multiple branched graft was constructed by suturing three grafts to the openings in a straight graft in an end-to-side fashion with running 4–0 polypropylene. Therefore, it comprised a main graft and three branches. The first branch was perpendicular to the other two branches, and the third branch was bifurcated into two limbs (Fig. 1).

**Fig. 2:**
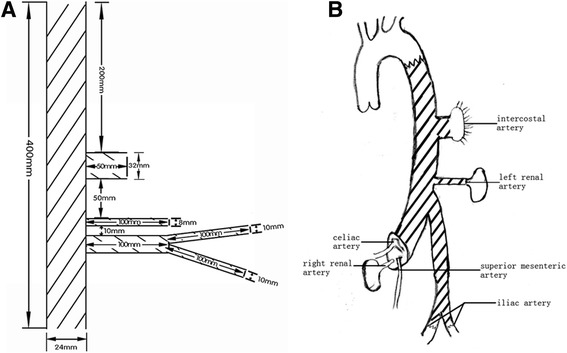
The sizes and attachment of the main graft and three branches. (**a**), Schematic drawing of all nastomoses (**b**)

The sizes and attachment of the main graft and three branches were showed in Fig. 2.

Patient was in the right lateral decubitus position with the lower body slightly tilted to the left, and a left thoracoabdominal incision was performed. The cardiopulmonary bypass was established by a venous cannula placed in the right atrium through the left femoral vein and 2 arterial return cannulas inside both femoral artery and ascending aorta. If the proximal thoracic descending aorta was not involved by the aneurysm and was long enough for both clamping and anastomosis (usually Crawford extent III TAAA), proximal aortic anastomosis was performed under mild hypothermic cardiopulmonary bypass and beating heart. otherwise, it was performed using an open technique with profound hypothermic circulatory arrest.

During our modified multiple branched graft replacement of TAAA, the proximal aortic anastomosis was performed first. After a proximal aortic clamp was placed just distal to the left subclavian artery or profound hypothermic circulatory arrest was established, a distal aortic clamp was positioned above the diaphragm. The aorta was transected proximal to the diseased segment and the proximal end of the choiced modified multiple branched graft was cut to the appropriate length. Then, the anastomosis between the proximal aorta and the proximal end of the modified multiple branched graft was performed with a continuous 4–0 polypropylene suture. As this proximal aortic anastomosis was completed, the air was evacuated from the graft, and a graft clamp was placed under this proximal aortic anastomosis.

Next, the distal aortic clamp was repositioned just above the ostia of the visceral arteries. The T6 to T12 intercostal arteries were isolated with a full-thickness patch of aorta. After this aortic patch was sutured to the first branch of the modified multiple branched graft, the graft clamp was released and replaced below the first branch to permit perfusion of those implanted intercostal arteries. During the graft clamp movement, air was evacuated from the graft. Then, the distal aortic clamp was repositioned above the aortic bifurcation. Orifices of celiac, superior mesenteric, and right renal arteries were joined at a single aortic patch, and this aortic patch was anastomosed to the distal end of the main graft. Thereafter, the graft clamp below the first branch was removed and air was evacuated from the graft, and both second and third branches were individually clamped. When this step was completed, perfusion to the viscera and right kidney was restored. The second branch was anastomosed to the left renal artery, and then the blood supply was restored to the left kidney after the second branch clamp removal and air evacuation Finally, the third branch was anastomosed to the normal distal abdominal aorta or it’s two limbs to the bilateral iliac arteries after the third branch or it’s limbs were trimmed in the suitable length.

## Discussion

**Fig. 3:**
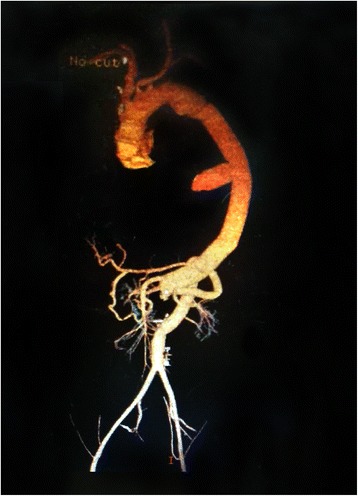
Postoperative computed tomographic scans showed that the thoracoabdominal aortic aneurysm was repaired with a modified multiple branched graft

From April 2009 to December 2014, the modified multiple branched graft was used in 8 patients with Crawford extent II (3 patients) and III (5 patients) TAAA (ie, 6 men and 2 woman; mean age 32.7 ± 9.4, range, 19 to 45 years). The cardiopulmonary bypass time, spinal cord ischemic time, visceral and right renal ischemic time and the left renal ischemic time were 176 ± 52 min (102–207 min), 16.3 ± 4.3 min (13–19 min), 16.5 ± 4.3 min (13–20 min), 25.8 ± 6.2 min (24–31 min), respectively. Performing all anastomotic hemostasis was easy as all the suture line was completely external exposed. No patient required a reopening to correct excessive post-procedural bleeding. Prolonged intubation (›48 h) was required in 2 patients. Renal failure requiring dialysis occurred only in 1 patient. Paraplegia or paraparesis did not develop postoperatively in any patient. No patient had clinical evidence for postoperative hepatic or intestinal ischemia. All were discharged from the hospital and were followed up. The follow-up period was 32.7 ± 9.9 months (range, 15–72 months). Their postoperative computed tomographic scans showed that all modified multiple branched grafts were patent and not kinked (Fig. 3). All of them survived well and had resumed normal activities.

Our modified multiple branched graft was specially designed for TAAA repair. It provided a branch for attachment to the vital intercostal arteries, and it’s all branches could easily conform to the different directions of the celiac, superior mesenteric, and renal arteries. Therefore, compared with the presewn tetrafurcate graft designed for arch replacement, our modified multiple branched graft might be more suitable for T AAA repair.

In the present technique, orifices of celiac, superior mesenteric, and right renal arteries were joined at a single aortic patch, and this aortic patch was anastomosed to the graft. Compared with the separate attachment technique in which each visceral or renal artery was anastomosed to the different branch of the multiple branched graft, our visceral patch technique could sensibly reduce the time for anastomosis and the ischemic time of viscera, consequently could simplify the operation. However, visceral patch aneurysm might develop. Fortunately, this visceral patch aneurysm didn’t occur in our patients during the follow-up. During the operation, we kept the residual aortic wall of the visceral patch as less as possible, which might contributed to our present results. Surely, the long-term fate of those visceral patches should be carefully evaluated, but the prevalence of patch aneurysm is expected to be acceptable because the straight graft and patch technique for TAAA repair provides satisfactory long-term fates of the visceral patches [3].

## Conclusions

Our present modified multiple branched graft may make TAAA replacement easier and safer. Although the preliminary outcomes were encouraging, rigorous long-term follow-up and further extensive clinical use are necessary to completely evaluate the efficacy of this modified multiple branched graft.

## Abbreviations

TAAA: Thoracoabdominal aortic aneurysm
